# Open reduction and internal fixation of a radius and ulna fracture in a patient with an elbow arthrodesis: a case report

**DOI:** 10.1080/23320885.2024.2378062

**Published:** 2024-07-09

**Authors:** Kyle Mangum, Taylor Blackwood, Tammam Hanna, Justin Harder, Evan Hernandez, Brendan MacKay

**Affiliations:** a School of Medicine, TX Tech University Health Sciences Center, Lubbock, TX, USA; b Department of Orthopaedic Surgery, Texas Tech University Health Sciences Center, Lubbock, TX, USA

**Keywords:** Titanium Elastic Nail System (TENS), elbow arthrodesis, Open reduction and internal fixation (ORIF)

## Abstract

This case report outlines the effective use of the Titanium Elastic Nail System (TENS) for treating a peri-implant mid-shaft radius and ulna fracture in a patient with previous elbow arthrodesis and rotational full-thickness flap coverage. Given the paucity of literature surrounding this complex problem, we present a minimally - invasive treatment option which facilitated complete fracture healing, demonstrating the TENS's efficacy in complex orthopedic scenarios.

## Introduction

Elbow arthrodesis (EA) is an uncommon surgery usually reserved as a last line treatment for challenging pathologies or as a salvage procedure [1]. The procedure involves the fixation of the elbow joint, with internal fixation using a posterior plate being preferred [1, 2]. Considering the niche indications for EA, patients who undergo this procedure are often medically complex and at high risk for complications [3, 4]. Due to the infrequent use of EA in the modern treatment of elbow pathology research on the topic is not extensive. This case report will discuss the successful treatment of a midshaft radius and ulna fracture sustained by a patient 2 years after their EA using a titanium elastic nail system (TENS).

## Case report

**Figure 1.**
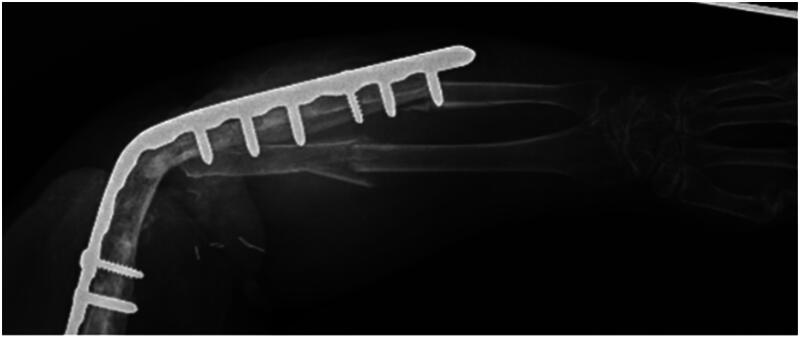
X-Ray taken prior to surgery. Midshaft fracture of the radius and ulna involving the elbow arthrodesis.

A 54-year-old female presented to the emergency department after a ground-level fall on her left elbow resulting in peri-implant midshaft radius and ulna fractures (Figure 1). The patient’s past medical history included hypertension, hyperlipidemia, osteopenia, anxiety, depression, chronic pain and a 25 pack-year history of tobacco use. This patient was well known to the orthopedic department for multiple complications resulting from a ground level fall with intra-articular left distal humerus fracture sustained six years prior. She had previously undergone multiple ORIF procedures, total elbow arthroplasty and eventually revision total elbow arthroplasty with multiple irrigation and debridement procedures requiring full-thickness rotational skin flap coverage and split-thickness skin grafting due to non-compliance, infection, and wound healing issues. Ultimately, the patient had last been seen approximately two years prior to this encounter and underwent uncomplicated elbow arthrodesis at that time with a 14-hole Synthes elbow arthrodesis plate. Given the patients significant previous history of complications and poor wound healing requiring rotational full-thickness flap coverage, the orthopedic team discussed the possible treatment options with the patient and a mutual decision was made for fixation of the fractures with a more conservative approach including partial removal of hardware and elastic nail implantation.

The patient was taken to the operating room and placed supine on the operating table with an arm table overlying the left side. The radial fixation was performed first using a 2.5 mm Synthes titanium elastic nail (TEN). A longitudinal incision was made over the radial side of the distal radius in standard fashion and the elastic nail was inserted through the medullary canal in standard retrograde fashion. The fracture was reduced with manual traction and supination, and the nail was driven across the fracture site under fluoroscopy. After confirming proper placement, attention was turned to the ulna.

The ulnar fracture site was identified under fluoroscopy and a longitudinal incision was made approximately 8 cm in length directly over this. The dissection was carried down to the fracture site through her previous elbow arthrodesis incision. The three distal most screws were removed from the elbow fixation hardware to allow for retrograde passage of the flexible nail. A 2.0 mm Synthes TEN was placed within the distal fracture site medullary canal and driven first in an antegrade fashion carefully through the distal ulna, and out the skin through a poke hole incision. The proximal medullary canal at the fracture site was then lightly reamed to allow passage of the nail as it was noted to have bony overgrowth within the medullary canal. The nail was driven in a retrograde fashion across the fracture site while the fracture was maintained and corrected into anatomical position using manual traction. There was still a small degree of gross motion noted at the fracture site and we elected to remove a third distal screw to improve the working length of the elastic nail. After this was performed and the nail was advanced, no gross motion was noted and relative stability had been achieved. With fixation complete, the pins were cut, and the bone tamp was used to prevent hardware irritation. At the conclusion of the case, the patient was placed into sterile dressings and a posterior slab splint.

**Figure 2.**
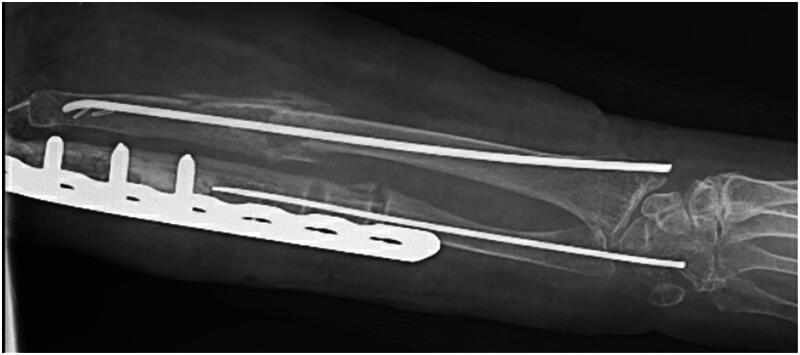
X-Ray 13 days following fracture fixation using TENS showing callus formation.

**Figure 3.**
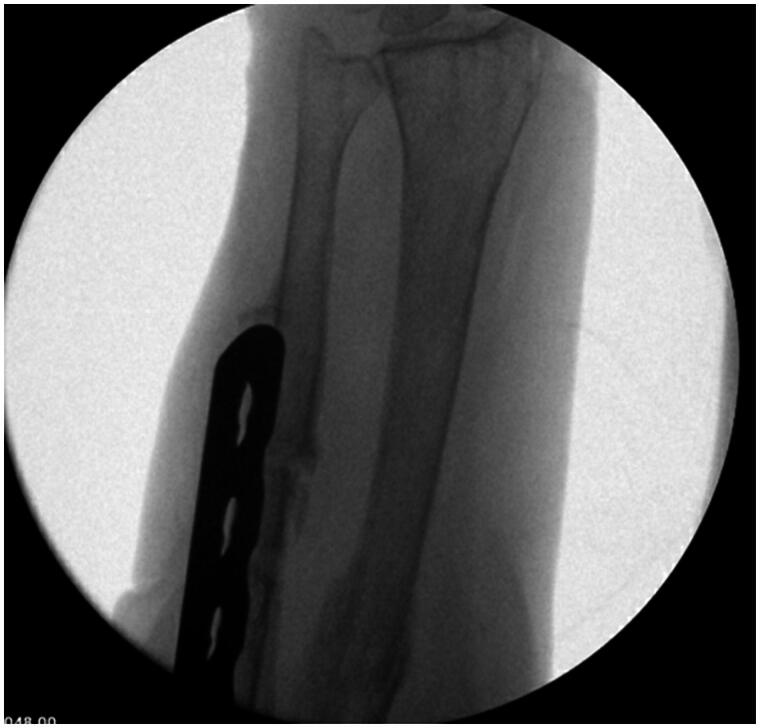
X-Ray taken following hardware removal showing healed fractures.

The patient’s post-operative course was uncomplicated. At two-week follow-up, her incisions had healed without any signs of infection. The patient was placed into a long arm cast for an additional five weeks. When her cast was removed at her seven-week post-operative visit, imaging was obtained which revealed abundant callous formation about the fracture sites with hardware in stable positioning (Figure 2). She was allowed to begin weight bearing at that time. The patient returned to clinic three months post-operatively to discuss hardware removal and imaging was obtained which demonstrated complete healing at the fracture site. Her hardware was removed in standard fashion approximately 16 weeks after placement with intraoperative stress radiographs at the fracture site demonstrating no motion and complete healing (Figure 3). The patient was lost to follow up 18 weeks post-operatively. During that appointment the patient demonstrated satisfactory range of motion with range of motion comparable to pre-injury status.

## Discussion

One of the main complications, outside of decreased ability to use the arm following EA, is fracture near the fixation site [2]. The creation of a long lever arm, elbow joint stiffness, and lack of pronosupination could all be factors that increase risk for fracture after EA [5]. These risks are greater in patients with decreased bone mass as seen in the patient presented above. While plate and screw fixation is still the gold standard for adult forearm fractures, TENS have been shown to be a viable alternative when indicated [6]. Intramedullary (IM) nails, compared to other means of ORIF, requires less soft tissue dissection and allow for easier hardware removal [7]. This makes TEN and other IM nail systems a promising choice in patients with a history of wound healing complications and infection like were present in this case. Other advantages of IM nails include lower risk of neurovascular injuries, soft tissue injuries, intraoperative fractures, muscle swelling, postoperative compartment syndrome and the load sharing properties of the implant [8–10]. IM nailing, especially in forearm fractures, comes with disadvantages as well. When compared to plate fixation, which provides absolute stability to support the forearm during healing, IM nails provide relative stability resulting in a frequent need for post-operative immobilization of the elbow, forearm and wrist until healing can occur [11]. Our patient was able to return to her pre-injury range of motion but stiffness is a concern with constructs which do not allow for early range of motion. In our case, callous formation was abundant early and attributable to the relative stability and minimal soft tissue disruption. An absolute stability construct may have led to increased soft tissue disruption and slower callous formation. Additionally, as TEN are elastic nails which provide less stability than rigid nails and ORIF, this can necessitate a prolonged period of limited weight bearing until fracture healing is evident. Despite these drawbacks, elastic IM nailing in this patient proved an effective method for achieving fracture healing while avoiding complications experienced previously by the patient. Our patient was relatively low demand and did not use her operative extremity for weight bearing more than five pounds. During discussion with the patient pre-operatively, her primary concern was her ability to get back to knitting and avoid infection which we felt a more minimally- invasive option with TENS versus ORIF accomplished. Further investigation into the use of IM nail systems in addressing fractures related to EA seems warranted, especially for low demand patients. In this case, the patient achieved complete union of the facture without infection or wound healing issues despite their complex medical and surgical history. The impact of the TENS system on this outcome is unknown and worth consideration. Due to the patient’s long history of delayed would healing, infections and eventual rotational flap and split-thickness skin grafting, the surgeon had concerns regarding revision elbow arthrodesis with a longer plate or with plate extensions due to the likely need for larger more invasive incisions. The radial shaft fracture was able to be fixed with a single percutaneous incision at the wrist and no disruption of the soft tissue and periosteum which lead to early callous formation. The ulnar shaft fracture necessitated open reduction as it was unable to be reduced with closed reduction due to the fracture site involving the most distal screw. During reduction, soft tissue and the two distal screws were removed which allowed for successful reduction. Operative fixation of fractures can have vast economic consequences for the patient and the healthcare system and should be considered when determining treatment plans. At our institution, the cost for a 2.0 and 2.5 mm TEN is $264.10 each, Making the estimated total for this case $528.20. The cost for a plate and screw construct for fracture fixation was estimated to total $816.12. While the financial impact was not a primary concern of the surgeon in operative planning, it does warrant consideration when choosing implant options with known equivalent outcomes. Given the paucity of literature surrounding this uncommon problem, the surgeons primary goal was to achieve fracture healing with minimal soft tissue disruption to allow the patient to return to her pre-injury level of function.

## Conclusion

This case demonstrates the successful use of a TENS to treat a midshaft fracture of the radius and ulna 2 years after an elbow arthrodesis. Not only was complete healing of the fracture achieved, but it was done so using a conservative, cost effective surgical approach which minimized potential complications and interference with existing hardware. Overall, this case report highlights the potential effectiveness of this approach for similar scenarios and emphasizes the importance of careful consideration of a patient’s individual health history and their desired outcomes. This case report adds to a topic that needs more research and reporting so outcomes can be improved in this complex patient population.
